# A feedback loop driven by H3K9 lactylation and HDAC2 in endothelial cells regulates VEGF-induced angiogenesis

**DOI:** 10.1186/s13059-024-03308-5

**Published:** 2024-06-25

**Authors:** Wei Fan, Shuhao Zeng, Xiaotang Wang, Guoqing Wang, Dan Liao, Ruonan Li, Siyuan He, Wanqian Li, Jiaxing Huang, Xingran Li, Jiangyi Liu, Na Li, Shengping Hou

**Affiliations:** 1 The First Affiliated Hospital of Chongqing Medical University, Chongqing Key Laboratory of Ophthalmology, Chongqing Eye Institute, Chongqing, China; 2 Department of Laboratory Medicine, Beijing Tongren Hospital, Capital Medical University, Beijing, 100005 China; 3 Beijing Institute of Ophthalmology, Beijing Tongren Eye Center, Beijing Tongren Hospital, Capital Medical University, Beijing Ophthalmology & Visual Sciences Key Laboratory, Beijing, 100730 China

**Keywords:** H3K9 lactylation, Angiogenesis, Endothelial cells (ECs), Histone deacetylase 2 (HDAC2), VEGF

## Abstract

**Background:**

Vascular endothelial growth factor (VEGF) is one of the most powerful proangiogenic factors and plays an important role in multiple diseases. Increased glycolytic rates and lactate accumulation are associated with pathological angiogenesis.

**Results:**

Here, we show that a feedback loop between H3K9 lactylation (H3K9la) and histone deacetylase 2 (HDAC2) in endothelial cells drives VEGF-induced angiogenesis. We find that the H3K9la levels are upregulated in endothelial cells in response to VEGF stimulation. Pharmacological inhibition of glycolysis decreases H3K9 lactylation and attenuates neovascularization. CUT& Tag analysis reveals that H3K9la is enriched at the promoters of a set of angiogenic genes and promotes their transcription. Interestingly, we find that hyperlactylation of H3K9 inhibits expression of the lactylation eraser HDAC2, whereas overexpression of HDAC2 decreases H3K9 lactylation and suppresses angiogenesis.

**Conclusions:**

Collectively, our study illustrates that H3K9la is important for VEGF-induced angiogenesis, and interruption of the H3K9la/HDAC2 feedback loop may represent a novel therapeutic method for treating pathological neovascularization.

**Supplementary Information:**

The online version contains supplementary material available at 10.1186/s13059-024-03308-5.

## Background

Vascular endothelial growth factor (VEGF) is an important regulator of blood vessel formation and function that controls several vital processes in endothelial cells (ECs), including proliferation, migration, and survival [1, 2]. Normal vascularization is essential for tissue development and organ function. However, dysregulation of vascularization is involved in multiple diseases, including proliferative diabetic retinopathy (PDR) [3], age-related macular degeneration (AMD) [4], retinopathy of prematurity (ROP) [5], and malignant tumor [6]. Anti-VEGF drugs are commonly used in clinical practice; however, their effects are sometimes limited because of drug insensitivity or resistance [7]. Elucidating the mechanisms underlying how VEGF stimulation influences the molecular and cellular processes of ECs and promotes neovascularization would help improve anti-angiogenesis therapies.

Previous studies have indicated that ECs are highly glycolytic, and metabolic reprogramming toward glycolysis is important for VEGF-induced EC behaviors [8, 9]. Glycolysis is the predominant bioenergetic pathway in ECs (approximately 85% of the total ATP is generated by glycolysis), and VEGF stimulation promotes glycolysis [9]. Genetic ablation of the glycolytic activator PFKFB3 in ECs greatly inhibits vessel formation both in vivo and in vitro [9]. Abundant glycolysis leads to lactate accumulation in ECs. Lactate has been defined as a metabolic byproduct. Recent studies have revealed the important functions of lactate in the regulation of multiple cellular processes, including T cell activation [10, 11], macrophage polarization [12] and tumor growth [13]. Whether and how the accumulated lactate influences EC behavior remain to be elucidated.

It is evidenced that histones play important roles in modulating chromatin structure and function [14]. Multiple post-translational modifications (PTMs), including acetylation, methylation, phosphorylation, and others, are powerful regulators of histone function and influence cellular processes that include transcriptional modification [14–16]. Lactate was recently identified as a substrate for a novel post-translational modification-lysine lactylation (Kla) [12]. Lactylation is a new method to transduce cellular metabolic characteristics into gene expression programs. Our studies and others have reported that lactylation can influence protein function and participate in multiple physiological and pathological processes [17–21]. The lactylation of H3K18 promotes the transcription of YTH N6-methyladenosine RNA-binding protein 2 (YTHDF2), which is involved in ocular melanoma [22]. The upregulation of H4K12 lactylation in microglia is important in AD pathogenesis [23]. We previously reported that lactylation of non-histone Ikzf1 at lysine 164 modulates Th17 cell differentiation and promotes autoimmune uveitis [19]. However, whether VEGF-induced glycolysis modulates ECs angiogenesis via lactylation remains unknown.

In the current study, we aimed to elucidate the role of glycolysis in angiogenesis. We found that upregulation of glycolysis promoted the lactylation of histone H3 at lysine residue 9 (H3K9la) in VEGF-stimulated ECs. Inhibition of lactylation efficiently suppressed angiogenesis. Mechanistically, CUT& Tag analysis revealed that H3K9 lactylation promoted the transcription of a set of genes related to angiogenesis. Furthermore, we identified a feedback loop between H3K9la and the Kla eraser HDAC2 in ECs that regulates angiogenesis. Collectively, our data link pathological angiogenesis with glycolysis-derived lactylation and suggest the presence of a feedback loop between H3K9la and HDAC2 in the angiogenesis pathway.

## Results

### H3K9 lactylation is increased in endothelial cells in response to VEGF stimulation

**Fig. 1:**
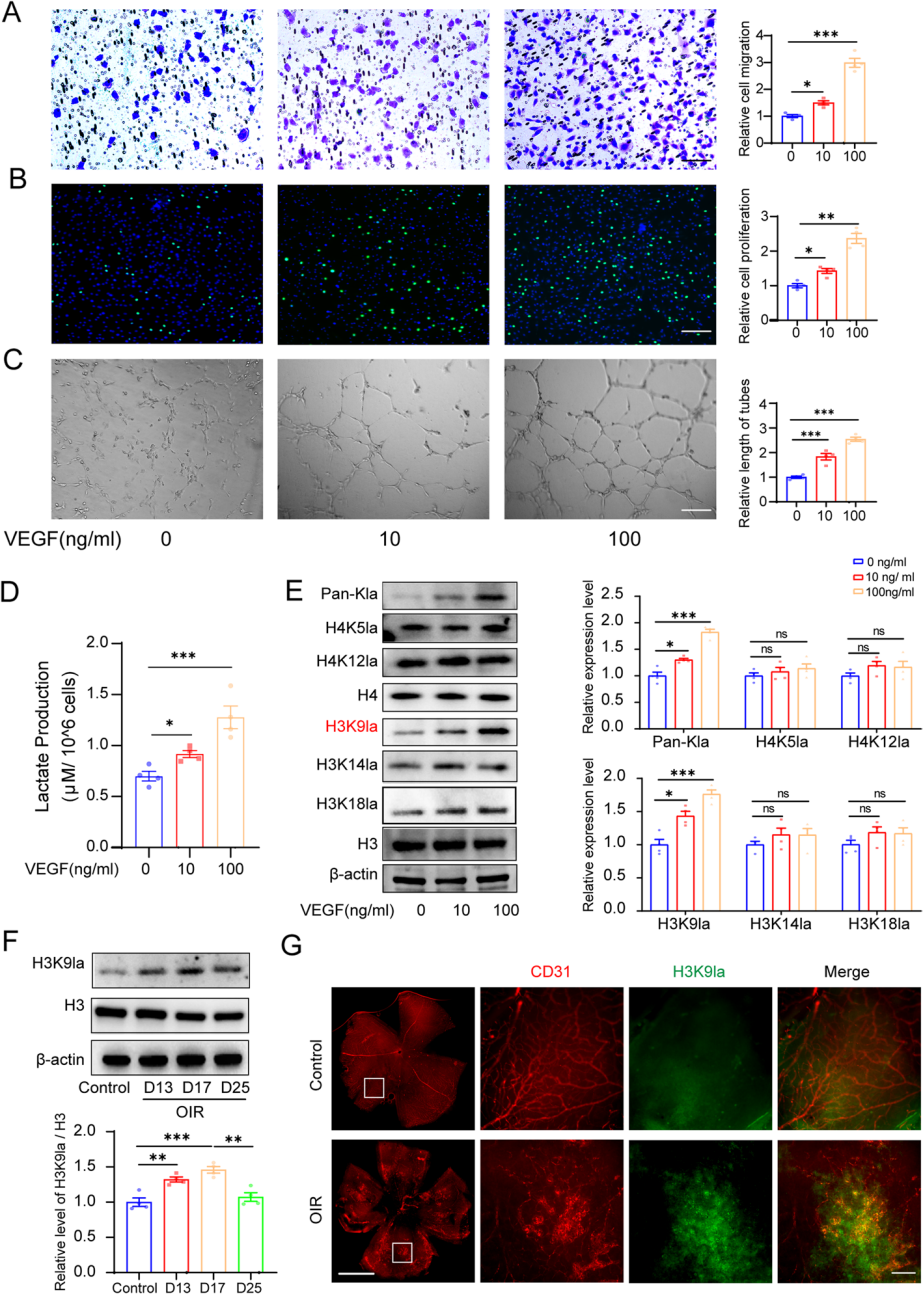
H3K9 lactylation is increased in endothelial cells in response to VEGF stimulation. **A** The effect of VEGF on migration of HRMECs (mean ± SEM; *n* = 4 samples per group; *P < 0.05, ***P < 0.001, one-way ANOVA, Bonferroni post hoc test; scale bars: 50 μm). **B** The effect of VEGF on proliferation of HRMECs (mean ± SEM; *n* = 4 samples per group; *P < 0.05, **P < 0.01, one-way ANOVA, Bonferroni post hoc test; scale bars: 100 μm). **C** The effect of VEGF on tube formation of HRMECs (mean ± SEM; *n* = 4 samples per group; ***P < 0.001, one-way ANOVA, Bonferroni post hoc test; scale bars: 50 μm). **D** The lactate content in HRMECs in response to VEGF stimulation (mean ± SEM; *n* = 4 samples per group; *P < 0.05, ***P < 0.001, one-way ANOVA, Bonferroni post hoc test). **E** Western blotting analysis of Pan-Kla and site-specific histone lactylation in HRMECs in response to VEGF stimulation (mean ± SEM; *n* = 4 samples per group; *P < 0.05, ***P < 0.001, ns: no significance, one-way ANOVA, Bonferroni post hoc test). **F** Western blotting analysis of H3K9la levels in OIR mice at different time points (mean ± SEM; *n* = 4 mice per group; **P < 0.01, ***P < 0.001, one-way ANOVA, Bonferroni post hoc test). **G** Representative images of H3K9la co-stained with endothelial cells (CD31) in normal and OIR mice retina (scale bars: left: 1000 μm; right: 50 μm)

Neovascularization is a complex process involving the activation, proliferation, and migration of endothelial cells [24, 25]. VEGF is the most powerful pro-angiogenic factor [25]. VEGF treatment promoted the proliferation, migration, and tube formation abilities of primary human retinal microvascular endothelial cells (HRMECs) in a dose-dependent manner (Fig. 1A–C). Lactate dehydrogenase A (LDHA) expression was increased and the lactate levels were upregulated in HRMECs in response to VEGF stimulation (Fig. 1D, Additional file 1: Fig. S1). We investigated whether upregulation of lactate content promoted histone lactylation in ECs. We analyzed the lactylation levels of a series of well-identified lactylated residues in histones, including H4K12la, H4K5la, H3K18la, H3K14la, and H3K9la. We observed that the lactylation level of H3K9 was significantly upregulated in response to VEGF stimulation (Fig. 1E). We examined the H3K9la levels in a mouse model of oxygen-induced retinopathy (OIR), a well-established mouse model of pathological retinal neovascularization. We found that the H3K9la increased with OIR progression and reached its peak at P17 (the peak of neovascularization in OIR) and decreased at day 25 (Fig. 1F). The H3K9la levels were elevated in the retinal endothelial cells of OIR mice compared to those in the age-matched controls (Fig. 1G). These results indicated that VEGF-induced angiogenesis was characterized by H3K9 lactylation and was likely to be involved in retinal neovascularization.

### Inhibition of H3K9la inhibited angiogenesis in vivo and in vitro

**Fig. 2:**
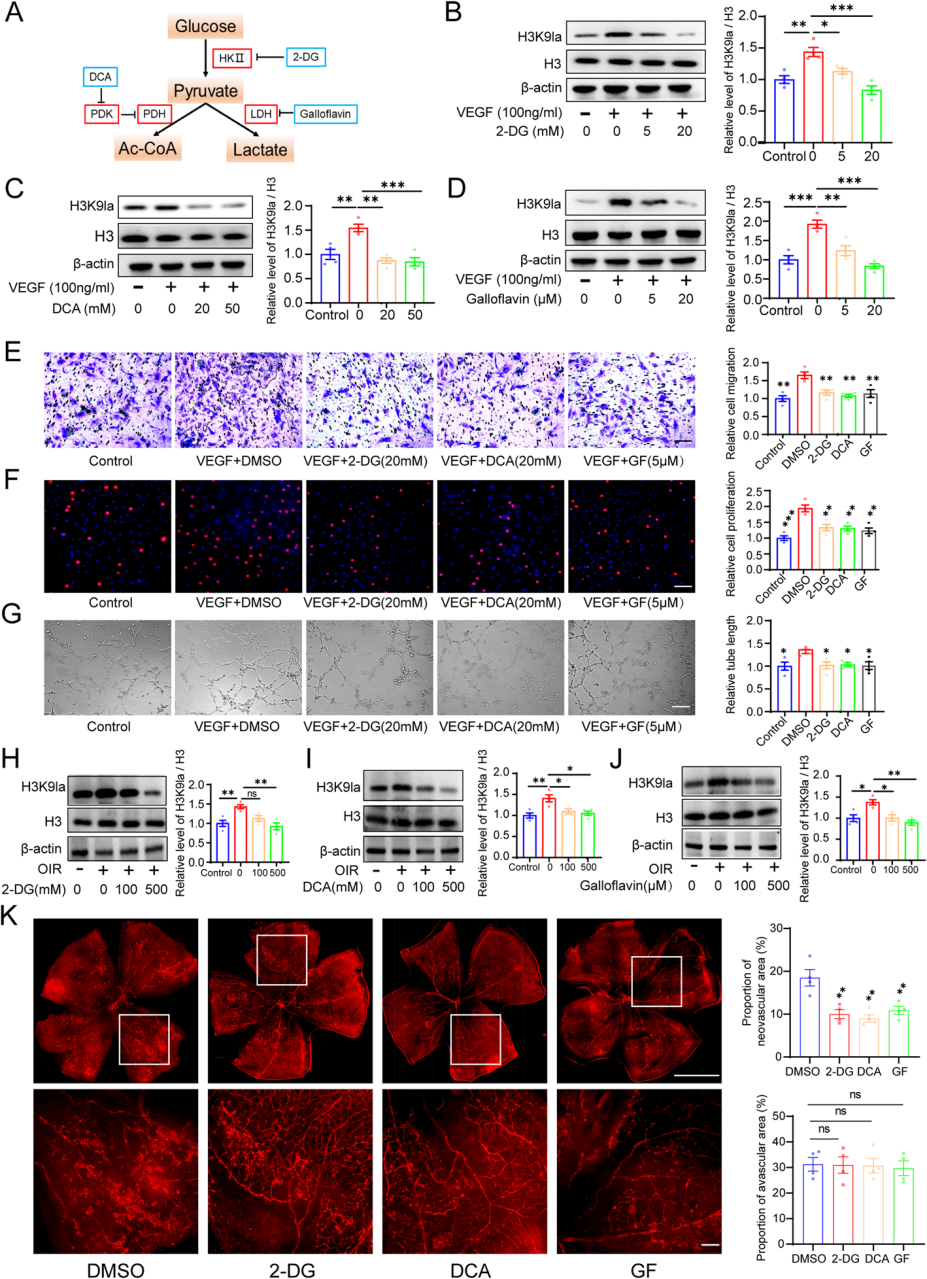
Inhibition of H3K9la inhibited angiogenesis in vivo and in vitro. **A** Schematic representation of treatment of HRMECs with glycolysis inhibitors. **B** Western blotting analysis of H3 and H3K9la levels in HRMECs in response to 2-DG treatment (mean ± SEM; *n* = 4 samples per group; *P < 0.05, **P < 0.01, ***P < 0.001, one-way ANOVA, Bonferroni post hoc test). **C** Western blotting analysis of H3 and H3K9la levels in HRMECs in response to DCA treatment (mean ± SEM; *n* = 4 samples per group; **P < 0.01, ***P < 0.001, one-way ANOVA, Bonferroni post hoc test). **D** Western blotting analysis of H3 and HE3K9la levels in HRMECs in response to galloflavin treatment (mean ± SEM; *n* = 4 samples per group; **P < 0.01, ***P < 0.001, one-way ANOVA, Bonferroni post hoc test). **E** The effect of glycolysis inhibitors on migration of HRMECs (mean ± SEM; *n* = 4 samples per group; **P < 0.01, one-way ANOVA, Bonferroni post hoc test, compared with DMSO group; scale bars: 20 μm). **F** The effect of glycolytic inhibitors on proliferation of HRMECs (mean ± SEM; *n* = 4 samples per group; **P < 0.01, ***P < 0.001, one-way ANOVA, Bonferroni post hoc test, compared with DMSO group; scale bars: 50 μm). **G** The effect of glycolytic inhibitors on tube formation of HRMECs (mean ± SEM; *n* = 4 samples per group; *P < 0.05, one-way ANOVA, Bonferroni post hoc test, compared with DMSO group; scale bars: 50 μm). **H** Western blotting analysis of H3K9la levels in OIR mice in response to 2-DG treatment (mean ± SEM; *n* = 4 mice per group; **P < 0.01, ***P < 0.001, ns: no significance, one-way ANOVA, Bonferroni post hoc test). **I** Western blotting analysis of H3K9la levels in OIR mice in response to DCA treatment (mean ± SEM; *n* = 4 mice per group; *P < 0.05, **P < 0.01, one-way ANOVA, Bonferroni post hoc test). **J** Western blotting analysis of H3K9la levels in OIR mice in response to galloflavin treatment (mean ± SEM; *n* = 4 mice per group; *P < 0.05, **P < 0.01, one-way ANOVA, Bonferroni post hoc test). **K** Representative images of retinal neovascularization in OIR mice in response to glycolysis inhibitors treatment (mean ± SEM; *n* = 4 mice per group; ns: no significance, **P < 0.01, ***P < 0.001, one-way ANOVA, Bonferroni post hoc test, compared with DMSO group; scale bars, up: 1000 μm; down: 100 μm)

Since lactate is the substrate for lactylation, we inhibited lactate production using glycolytic inhibitors to examine whether the inhibition of histone lactylation could suppress angiogenesis. As previously reported, the glycolytic inhibitors used include the non-metabolizable glucose analog-2-deoxy-d-glucose (2-DG), the pyruvate dehydrogenase kinase (PDK) inhibitor- dichloroacetate (DCA), and the lactic acid dehydrogenase (LDH) inhibitor-galloflavin (GF) (Fig. 2A) [12, 22, 26]. We found that these inhibitors caused a notable decrease in cellular lactate production and H3K9 lactylation (Fig. 2B–D, Additional file 1: Fig. S2A-C). Notably, following a decrease in the H3K9 lactylation levels, the migration, proliferation, and tube formation abilities of ECs were significantly suppressed (Fig. 2E–G). We then applied inhibitors treatment in OIR mice. Consequently, we found that inhibitors treatment decreased H3K9 lactylation in vivo and alleviated pathological neovascularization of OIR mice, but it did not influence the proportion of avascular area (Fig. 2H–K). In conclusion, the above results suggest that H3K9 lactylation is important for angiogenesis and inhibition of H3K9 lactylation may represent an efficient way to suppress neovascularization.

### Identification of potential downstream targets of H3K9 lactylation by genome-wide CUT& Tag analysis

**Fig. 3:**
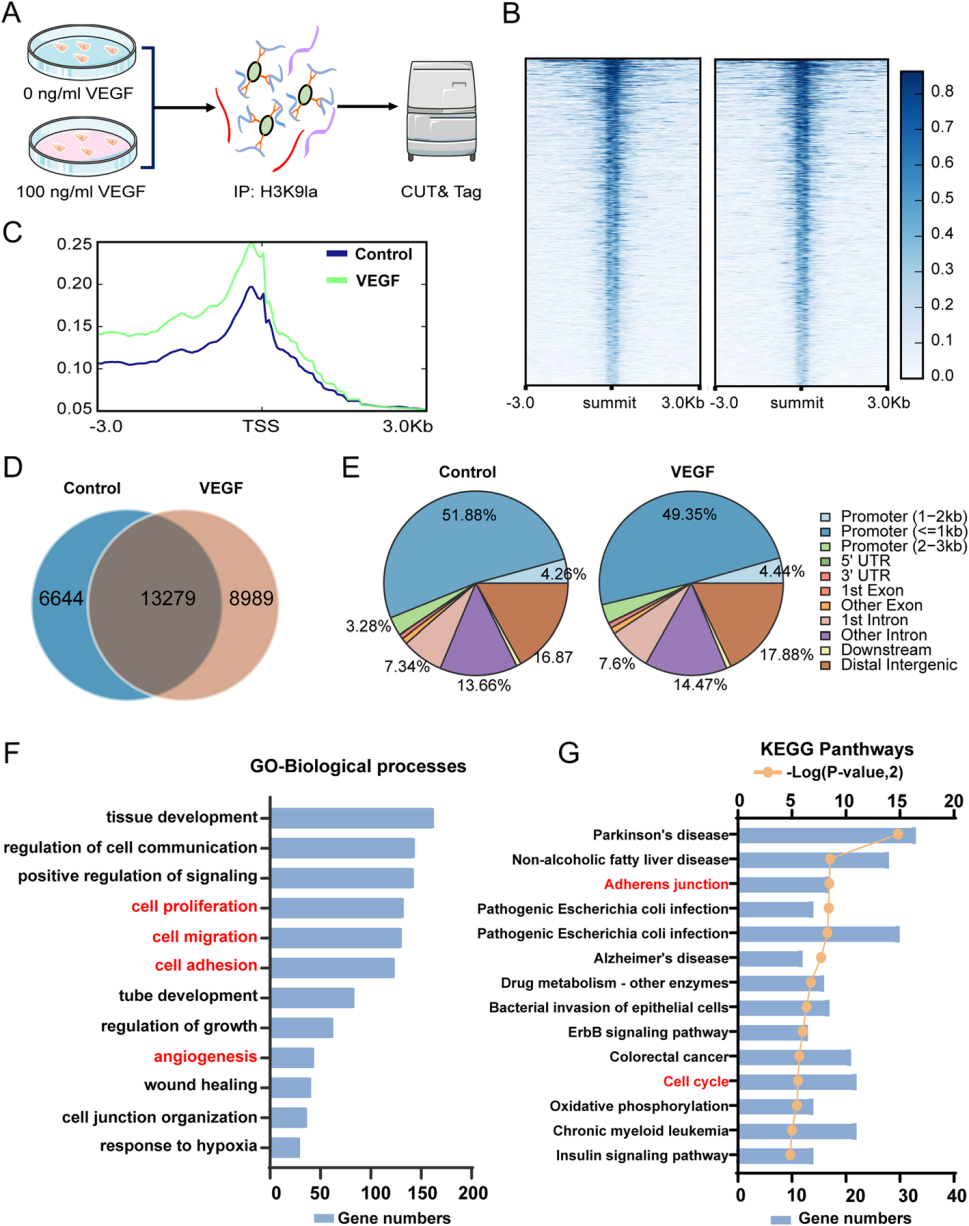
Identification of potential downstream targets of H3K9 lactylation by genome-wide CUT& Tag analysis. **A** Schematic representation of CUT& Tag analysis. **B** The binding density of H3K9la in endothelial cells was visualized by deepTools. **C** Line chart of reads distribution relative to TSS. **D** The number of peaks identified in control and VEGF groups. **E** Genome-wide distribution of H3K9la-binding peaks in endothelial cells. **F** GO analysis of the elevated H3K9la binding peaks at candidate target genes. **G** KEGG analysis of the elevated H3K9la binding peaks at candidate target genes

Histone modifications play an important role in regulating the transcription of target genes [23, 27]. Genome-wide CUT& Tag analysis is a newly described method for studying protein-DNA interactions [28]. Therefore, we performed CUT& Tag analysis to identify the candidate genes regulated by H3K9la in ECs (Fig. 3A). Briefly, HRMECs were treated with 0 or 100 ng/ml VEGF for 24 h. CUT& Tag analysis was performed using antibodies against H3K9la, and analysis of the results with deep tools revealed enrichment of H3K9la peaks in ECs (Fig. 3B,C). The results showed that 13,279 H3K9la binding peaks were identified in both groups and over 55% of the binding peaks were located in promoter sequences (< 3 kb) (Fig. 3D, E). Pearson’s correlation analysis revealed a significant correlation between the two groups (Additional file 1: Fig. S3) and the identified peaks were evenly distributed across all chromosomes (Additional file 1: Fig. S4). To analyze the epigenetic modulatory effects of H3K9la in ECs in response to VEGF stimulation, the target genes with differential binding peaks (fold change < 0.67 or > 1.5) were classified by gene ontology (GO) and Kyoto Encyclopedia of Genes and Genomes (KEGG) analyses. Interestingly, we found that the upregulated peak-related genes were enriched in multiple pathways related to neovascularization, including cell proliferation, cell migration, adherens junction, and angiogenesis (Fig. 3F, G).

### H3K9 lactylation activates the transcription of multiple genes related to angiogenesis

**Fig. 4:**
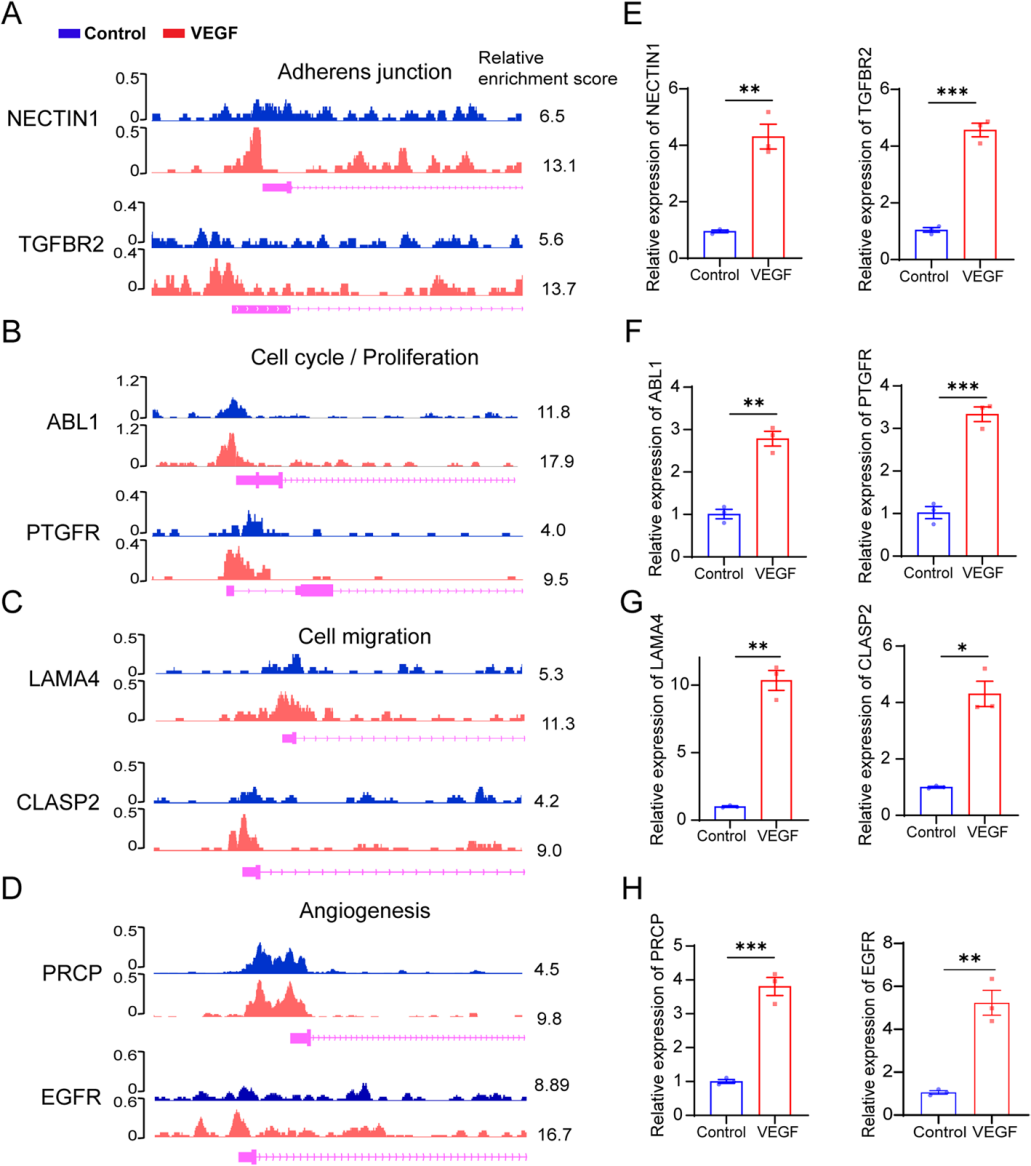
H3K9 lactylation activates the transcription of multiple genes related to angiogenesis. **A** Genome browser tracks of CUT& Tag signal at the NECTIN1 and TGFBR2 loci. **B** Genome browser tracks of CUT& Tag signal at the ABL1 and PTGFR loci. **C** Genome browser tracks of CUT& Tag signal at the LAMA4 and CLASP2 loci. **D** Genome browser tracks of CUT& Tag signal at the PRCP and EGFR loci. **E** qPCR assays monitoring expression of NECTIN1 and TGFBR2 in HRMECs treated with or without VEGF (mean ± SEM; *n* = 3 samples per group; **P < 0.01, unpaired Student’s *t* test). **F** qPCR assays monitoring expression of ABL1 and PTGFR in HRMECs treated with or without VEGF (mean ± SEM; *n* = 3 samples per group; **P < 0.01, ***P < 0.001, unpaired Student’s *t* test). **G** qPCR assays monitoring expression of LAMA4 and CLASP2 in HRMECs treated with or without VEGF (mean ± SEM; *n* = 3 samples per group; *P < 0.05, **P < 0.01, unpaired Student’s *t* test). **H** qPCR assays monitoring expression of PRCP and EGFR in HRMECs treated with or without VEGF (mean ± SEM; *n* = 3 samples per group; ***P < 0.001, unpaired Student’s *t* test)

Considering that the lactylation level of H3K9la was upregulated in VEGF-stimulated ECs and that hyperlactylated H3K9 was enriched in genes related to angiogenic pathways, we further investigated whether the expression of these genes was elevated in response to VEGF stimulation. Specifically, CUT& Tag analysis revealed that the levels of H3K9la were upregulated at the promoters of candidate genes in VEGF-stimulated HRMECs, including NECTIN1 [29], TGFBR2 [30], ABL1 [31], PTGFR [32], LAMA4 [33], CLASP2 [34], PRCP [35], and EGFR [36] which are related to above interested pathways (Fig. 4A–D). Chromatin immunoprecipitation qPCR (CHIP-qPCR) analysis confirmed that hyperlactylation promoted H3K9la’s binding to the promoters of these genes (Additional file 1: Fig. S5A-B). Furthermore, qPCR assays confirmed that the expression levels of these genes were significantly upregulated in VEGF stimulated ECs (Fig. 4E–H). Collectively, these results indicate that H3K9 lactylation may participate in angiogenesis by promoting the expression of multiple genes encoding pro-angiogenic proteins in response to VEGF stimulation.

### Feedback loop driven by H3K9 lactylation and HDAC2 promotes angiogenesis

**Fig. 5:**
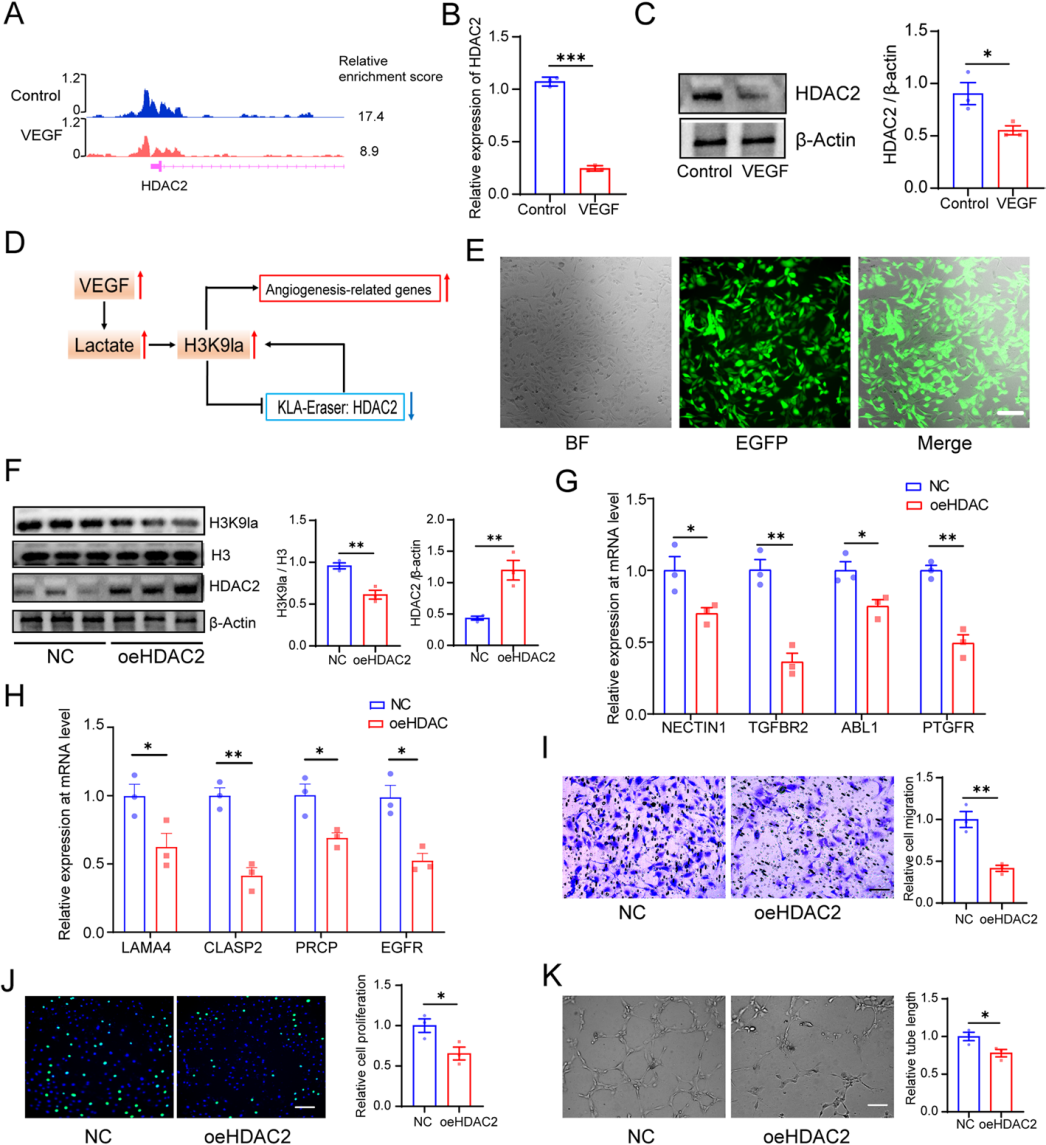
A feedback loop driven by H3K9 lactylation and HDAC2 promotes angiogenesis. **A** Genome browser tracks of CUT& Tag signal at the HDAC2 loci. **B** qPCR assays monitoring expression of HDAC2 in HRMECs treated with or without VEGF (mean ± SEM; *n* = 3 samples per group; ***P < 0.001, unpaired Student’s *t* test). **C** Western blotting analysis of HDAC2 levels in HRMECs treated with or without VEGF (mean ± SEM; *n* = 3 samples per group; *P < 0.05, unpaired Student’s *t* test). **D** Schematic of the feedback loop driven by H3K9 lactylation and HDAC2. **E** Transfection efficiency of the oeHDAC2 lentivirus in HRMECs (BF, bright field; EGFP, enhanced green fluorescence protein; scale bar, 20 μm). **F** Western blotting analysis of HDAC2 and H3K9la levels in HRMECs overexpressing HDAC2 or not (mean ± SEM; *n* = 3 samples per group; **P < 0.01, unpaired Student’s *t* test). **G** qPCR assays monitoring expression of NECTIN1, TGFBR2, ABL1, and PTGFR in HRMECs overexpressing HDAC2 or not (mean ± SEM; *n* = 3 samples per group; *P < 0.05, **P < 0.01, unpaired Student’s *t* test). **H** qPCR assays monitoring expression of LAMA4, CLASP2, PRCP, and EGFR in HRMECs overexpressing HDAC2 or not (mean ± SEM; *n* = 3 samples per group; *P < 0.05, **P < 0.01, unpaired Student’s *t* test). **I** The effect of overexpressing HDAC2 on migration of HRMECs (mean ± SEM; *n* = 3 samples per group; **P < 0.01, unpaired Student’s *t* test; scale bars: 20 μm). **J** The effect of overexpressing HDAC2 on proliferation of HRMECs (mean ± SEM; *n* = 3 samples per group; *P < 0.05, unpaired Student’s *t* test; scale bars: 50 μm). **K** The effect of overexpressing HDAC2 on tube formation of HRMECs (mean ± SEM; *n* = 3 samples per group; *P < 0.05, unpaired Student’s *t* test; scale bars: 20 μm)

Interestingly, CUT& Tag analysis revealed a decrease in levels of H3K9la at the promotors of HDAC2 in VEGF-stimulated ECs (Fig. 5A). And we observed a decrease in the expression of HDAC2 at both the mRNA and protein levels in VEGF-stimulated ECs (Fig. 5B,C). CHIP-qPCR assays also confirmed that hyperlactylation of H3K9 decreased its binding to the promoters of HDAC2 and this process is reversed in response to glycolysis inhibitor treatment (Additional file 1: Fig. S5 C-D). A previous study reported that HDAC2 is an effective lactylation eraser [37]. We hypothesized that there existed a feedback loop in which VEGF stimulation increased H3K9 lactylation and then inhibited HDAC2 expression; decreased HDAC2 expression in turn promoted H3K9 lactylation and elevated the expression of the target angiogenic genes (Fig. 5D). To confirm the functional impact of the VEGF/H3K9la/HDAC2 feedback loop on ECs, we overexpressed HDAC2 in VEGF-stimulated ECs using lentivirus. The transfection efficiency was over 90% (Fig. 5E). We observed that overexpression of HDAC2 resulted in significant decrease in H3K9 lactylation (Fig. 5F). Consistent with our previous results showing that H3K9la activated gene expression, we found that the levels of these angiogenic genes decreased in HDAC2-overexpressing ECs (Fig. 5G,H). Meanwhile, HDAC2 overexpression inhibited EC angiogenesis (Fig. 5I–K). Taken together, these results revealed that HDAC2 overexpression disrupted the VEGF/ H3K9la/ HDAC2 feedback loop in ECs and suppressed angiogenesis.

## Discussion

**Fig. 6:**
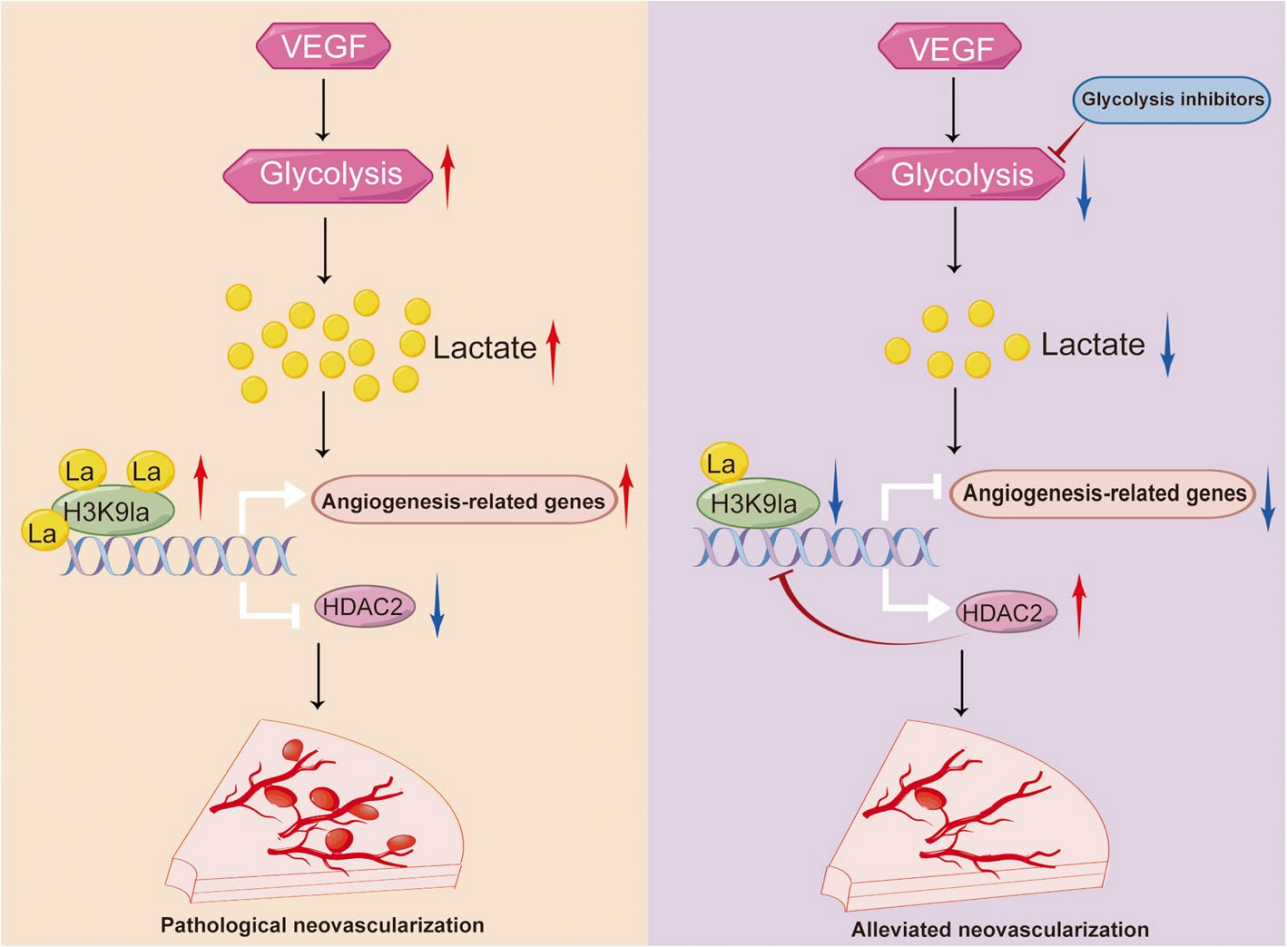
A proposed working model for how a glycolysis/H3K9la/HDAC2 feedback loop in endothelial cells modulates angiogenesis (By Figdraw)

Histone lactylation is a recently identified lactate-dependent epigenetic modification [12], which represents a novel mechanism for the transduction of cellular metabolic patterns into gene expression programs via activation or inhibition of gene transcription. Lactylation plays important roles in multiple cellular processes, including the modulation of macrophage M1/2 polarization [12], exacerbation of microglial dysfunction [23], and promotion of tumorigenesis [27]. In this study, we discovered a feedback loop driven by H3K9la and HDAC2 in ECs promoted angiogenesis in response to VEGF stimulation. VEGF treatment increased H3K9 lactylation levels and promoted its binding to the promoters of a set of angiogenesis-related genes. Treatment with glycolytic inhibitors or HDAC2 overexpression decreased H3K9 lactylation levels and suppressed angiogenesis. Collectively, we analyzed the mechanistic link between VEGF stimulation, histone lactylation and angiogenesis and suggested potential therapeutic approaches for pathological neovascularization (Fig. 6).

Numerous studies have shown that cellular metabolism is not only important for cell survival but can also influence cell behaviors [38–40]. Glycolysis plays an important role in EC angiogenesis and results in elevated lactate levels [9]. However, the role of lactate in regulating neovascularization remains largely unknown. Metabolic byproducts are commonly sources for post translational modifications and affects protein function. Our results showed that lactate contributes to angiogenesis by increasing H3K9 lactylation and promoting pro-angiogenic transcriptional programs in ECs. Inhibition of lactate production with glycolysis inhibitors reduced H3K9 lactylation levels and suppressed neovascularization. These results revealed a novel mechanism by which lactate promotes angiogenesis by modulating histone lactylation.

VEGF is a predominant proangiogenic factor that activates endothelial cells [1]. Histone lactylation is known to function in regulating gene transcription [17, 22]. Therefore, we conducted CUT& Tag analysis to identify the target genes regulated by H3K9 lactylation in response to VEGF stimulation. CUT& Tag analysis is an enzyme-tethering strategy that provides efficient high-resolution sequencing libraries for profiling diverse chromatin components [28]. Our results showed that H3K9la was highly enriched in gene promoters and was correlated with the activation of genes associated with angiogenesis. Neovascularization is a complex process. We found that the upregulated H3K9la-binding genes were enriched in pathways related to angiogenesis, including cell proliferation, cell migration, and adherens junction. These results further demonstrated the important role of H3K9la in angiogenesis.

Similar to the regulatory mechanism of other post-translational modifications, lactylation is regulated by key regulatory enzymes, including lactyltransferases and delactylases. HDAC2 has been previously identified as an efficient delactylase [35]. In the present study, we observed a decrease in HDAC2 expression levels in ECs in response to VEGF stimulation. Interestingly, CUT& Tag analysis revealed a decrease in H3K9la enrichment in the promoters of HDAC2. Therefore, we hypothesized that a feedback loop existed between H3K9la and HDAC2. As previous studies reported, histones play important roles in modulating chromatin structure and function, including transcriptional regulation [14, 15]. Post-translational modifications could influence histone’s binding to the promoters of specific genes and then regulates gene transcription [20, 21]. We found that hyperlactylation of H3K9 inhibited its interaction with HDAC2, and this process is reversed in response to glycolysis inhibitor treatment. Notably, HDAC2 overexpression decreased H3K9 lactylation levels and suppressed the transcription of the downstream target genes of H3K9la. Meanwhile, HDAC2 overexpression inhibits the angiogenic abilities of endothelial cells. Therefore, we think that lactylation regulates H3K9’s binding to the promoters of HDAC2 and modulates its expression. These results implicated HDAC2 as a potential therapeutic target for pathological neovascularization.

However, there are several limitations of our study. First, we proved that overexpression of HDAC2 decreased H3K9 lactylation and inhibited angiogenesis in vitro. Further studies such as knocking in HDAC2 in endothelial cells are needed to verify these results in vivo. Secondly, our results showed that hyperlactylation of H3K9 promoted angiogenic gene expression, including TGFBR2, PRCP, and EGFR. However, the transcriptional regulation of other classical angiogenesis genes such as VEGFR and FGFR needs further investigation.

## Conclusions

In conclusion, our results demonstrate that upregulated glycolysis promotes H3K9 lactylation in endothelial cells in response to VEGF stimulation, and we investigate the potential target angiogenic genes that undergo H3K9 lactylation. Meanwhile, we investigate the feedback loop between H3K9la and Kla eraser HDAC2 in ECs that regulates angiogenesis. Disruption of the H3K9la/HDAC2 feedback loop represents a novel method for treating pathological neovascularization.

## Methods

### Animals

C57BL/6 J mice were used for conducting all animal experiments in this study. The mice were housed at the Experimental Animal Center of Chongqing Medical University (Chongqing, China). All experiments were approved by the Ethics Committee of the First Affiliated Hospital of Chongqing Medical University (approval number: 2021–612).

### Induction of OIR

OIR was induced as previously described using an oxygen chamber (Chenxi Dianzi; Zhejiang; China) [41]. On the postnatal day 7 (P7), mouse pups and suckling mice were placed in the chamber with the oxygen concentration maintained at 75 ± 2% for five consecutive days. On P12, the pups were returned to room air (21% O_2_) and continually co-bred with the suckling mice for 5 days. Control pups were maintained in ambient air throughout the experiment. The retinas were extracted for subsequent experiments on P17. Glycolysis inhibitors were diluted in 0.1% DMSO and one microliter of each was administered by intravitreal injection at P13.

### Cell lines and cell cultures

HRMECs were purchased from Cell Systems (ACBRI 181, Seattle, America) and cultured with EGM™-2 Endothelial Cell Growth Medium-2 BulletKit (C3162, Lonza). Recombinant human VEGF were purchased from R&D systems (BT-VEGF-020).

### Migration assay

HRMECs (1 × 10^4)^ were seeded into the upper chambers of 24-well Transwell chambers with 8-μm pore size filters (3422, Corning) and cultured under corresponding conditions for 24 h. After fixation with 4% paraformaldehyde and stained with 1% crystal violet, the migrated cells were imaged under a microscope (Leica, Germany) and the number of cells were counted using ImageJ software. Four random images for each well were captured and the average value is used for statistical analysis.

### Tube formation assay

HRMECs were pretreated under different conditions for 18 h. We precoated 96-well plates with 50 µl of Matrigel (3432–005-01, R&D systems) for 40 min at 37 ℃. Next, a total of 1 × 10^4^ HRMECs resuspended in 100 µL of the corresponding medium were seeded per well and cultured for 6 h. Four random images for each sample were captured using a microscope (Leica, Germany) and the tube length was measured using ImageJ software.

### Proliferation assay

HMREC proliferation was assayed using an EdU Cell Proliferation Kit (C0071S, Beyotime) according to the manufacturer’s instructions. Briefly, HRMECs (4 × 10^4^) were seeded in 24-well plates and pretreated for 24 h. Then half of the medium was replaced with EdU buffer (20 μM) and incubated for another 2 h. After fixation and permeabilization, the cells were stained with click reaction solution and DAPI (1:1000). Four random images for each well were captured and the average value is used for statistical analysis.

### RT-qPCR

RNA was extracted from HRMECs using the Total RNA Isolation Kit (Vazyme, RC112-01). The qRT-PCR assays were performed using a PrimeScript RT–PCR kit (RR014A, Takara, Japan) on ABI 7500 Prism system (Applied Biosystems, USA). β-actin was used as internal control. The primers used are listed in Additional file 2: Table S1 and were purchased from Shanghai Sangon Co. Ltd (Shanghai, China).

### Western blotting

Tissue and cell samples were lysed with RIPA lysis buffer (Beyotime, P0013B). Proteins were separated using SDS-PAGE and transferred onto PVDF membranes (Millipore, MA, USA). The membranes were incubated with primary antibodies at 4 ℃ overnight and then with HRP-conjugated secondary antibodies at room temperature for 1 h. The ECL kit (K-12045-D20, Advansta, CA, USA) was used to visualize the signals. Data were analyzed using ImageJ software and normalized to the control group. The primary antibodies used for western blotting were as follows: Anti-Histone H3 (Abcam, ab18521, diluted 1:1000); anti-Histone H4 (CST, 2592, diluted 1:1000); anti-H3K9la (PTM BIO, PTM-1419RM, diluted 1:1000); anti-H3K14la (PTM BIO, PTM-1414RM, diluted 1:1000); anti-H3K18la (PTM BIO, PTM-1427RM, diluted 1:1000); anti-H4K5la (PTM BIO, PTM-1407, diluted 1:1000); anti-H4K12la (PTM BIO, PTM-1411RM, diluted 1:1000); anti-HDAC2 (CST, 57,156, diluted 1:1000); anti-β-Actin (Affinity, AF7018, diluted 1:3000); secondary antibody: Goat Anti-Rabbit IgG (H + L) HRP (Affinity, S0001, diluted 1:5000).

### Immunofluorescence

The retinal stretched preparations were permeabilized with 0.4% Triton X-100 for 30 min, blocked with 2% BSA for 1 h and incubated with the primary antibodies (Abcam, ab9498, 1:1000) for 12 h at 4 °C. The samples were then incubated with secondary antibodies at room temperature for 1 h. Images were captured using a confocal microscope (Leica, Germany). The following primary antibodies were used: CD31 (Abcam, ab9498, diluted 1:1000); H3K9la (PTM-1419RM, diluted 1:100).

### Quantification of lactate levels

The lactate concentration was assayed using an LA Content Assay Kit (Solarbio, BC2235) according to the manufacturer’s instructions. The signals were detected using a Varioskan LUX Microplate reader (Thermo Fisher Scientific).

### In vitro lentivirus infection

HRMECs were seeded in 6-well-plates at a density of 1 × 10^5^ cells per well overnight and then transfected with lentiviruses to overexpress HDAC2 (Shanghai Genechem Co, Ltd) at an MOI of 30. Three days later, transfection efficiency was determined using a fluorescence microscope (Leica, Germany). After selection with puromycin for 3 days, the cells were used for further experiments.

### CHIP assay

The CHIP assays were conducted using the Simple ChIP® Enzymatic Chromatin IP Kit (9003, CST). A total of 4 × 10^6^ HRMECs for each sample were crosslinked with formaldehyde (CST, 12,606) at final concentration of 1% for 5 min at room temperature and then quenched with 125 mM glycine (7005, CST). The nuclei preparation and chromatin immunoprecipitation procedures were conducted according to the manufacturer’s instructions using IgG (2729, CST) and H3K9la (PTM BIO, PTM-1419RM) antibodies. Then the qPCR assays were conducted using the ABI 7500 Prism system (Applied Biosystems, USA). The primers used are listed in Additional file 2: Table S2 and were purchased from Shanghai Sangon Co. Ltd (Shanghai, China).

### CUT& Tag

CUT& Tag was performed by Novogene Co., Ltd (Beijing, China) using the Hyperactive Universal CUT& Tag Assay Kit for Illumina (Vazyme). Briefly, the HRMECs were bound to concanavalin A-coated beads. After permeabilization and incubation with primary antibody (anti-H3K9la, PTM BIO, PTM-1419RM), the DNA was precisely tagmented with pA-Tn5 transposase and enriched by PCR to create sequencing-ready libraries. The library preparations were sequenced using the Illumina Novaseq platform, and 150 bp paired-end reads were generated.

### Quantification and statistical analysis

Statistical analyses were performed using unpaired Student’s *t* test or one-way ANOVA, Bonferroni post hoc test as indicated in the figure legends using SPSS (version 20.0). Shapiro–Wilk test was used to test the normality of data. Bar graphs represent the mean ± SEM. Statistical significance was set at *P* < 0.05.

### Supplementary Information

Additional file 1: Fig S1. H3K9 lactylation is increased in endothelial cells in response to VEGF stimulation. Fig S2. Inhibition of H3K9la inhibited angiogenesis in vivo and in vitro. Fig S3. Pearson correlation of fold enrichment between two groups. Fig S4. Location of identified peaks in chromosomes. Fig S5. A feedback loop driven by H3K9 lactylation and HDAC2 promotes angiogenesis.

Additional file 2: Table S1 and Table S2. Primers used in this study.

Additional file 3. Uncropped blot images.

Additional file 4. Review history.

## Data Availability

The CUT& Tag data are available in the Gene Expression Omnibus with accession number: GSE248643 [42]. No other scripts and software were used other than those mentioned in the “ Methods” section.
