# Supplementary material for: A feedback loop driven by H3K9 lactylation and HDAC2 in endothelial cells regulates VEGF-induced angiogenesis
Source: Genome Biol. 2024 Jun 25;25:165. doi: 10.1186/s13059-024-03308-5 (PMC11197246; doi:10.1186/s13059-024-03308-5)
Supplement: Supplementary file 1 — Additional file 1: Fig S1. H3K9 lactylation is increased in endothelial cells in response to VEGF stimulation. Fig S2. Inhibition of H3K9la inhibited angiogenesis in vivo and in vitro. Fig S3. Pearson correlation of fold enrichment between two groups. Fig S4. Location of identified peaks in chromosomes. Fig S5. A feedback loop driven by H3K9 lactylation and HDAC2 promotes angiogenesis. [file 13059_2024_3308_MOESM1_ESM.pdf]

Supplementary materials for:

A feedback loop driven by H3K9 lactylation and HDAC2 in endothelial cells regulates VEGF-induced angiogenesis

Authors： Wei Fan[1, #], Shuhao Zeng[1, #], Xiaotang Wang[1, #], Guoqing Wang[1], Dan Liao[1], Ruonan Li[1], Siyuan He[1], Wanqian Li[1], Jiaxing Huang[1], Xingran Li[1], Jiangyi Liu[1], Na Li[2, *], and Shengping Hou[1,3, *]

Affiliations

[1]The First Affiliated Hospital of Chongqing Medical University, Chongqing Key Laboratory of Ophthalmology, Chongqing Eye Institute, Chongqing, China

[2]Department of Laboratory Medicine, Beijing Tongren Hospital, Capital Medical University, Beijing, 100005, China.

[3]Beijing Institute of Ophthalmology, Beijing Tongren Eye Center, Beijing Tongren Hospital, Capital Medical University, Beijing Ophthalmology & Visual Sciences Key Laboratory, Beijing, 100730, China.

[#]These authors contributed equally.

*Corresponding author:

Shengping Hou Ph.D, The First Affiliated Hospital of Chongqing Medical University, Chongqing 400016, China; Beijing Institute of Ophthalmology, Beijing Tongren Eye Center, Beijing Tongren Hospital, Capital Medical University, Beijing Ophthalmology & Visual Sciences Key Laboratory, Beijing, 100730, China. E-mail address: sphou828@163.com; Telephone number: +86-10-58265906

Na Li Ph.D, Department of Laboratory Medicine, Beijing Tongren Hospital, Capital Medical University, Beijing, 100005, China; E-mail address: coco0411@126.com;

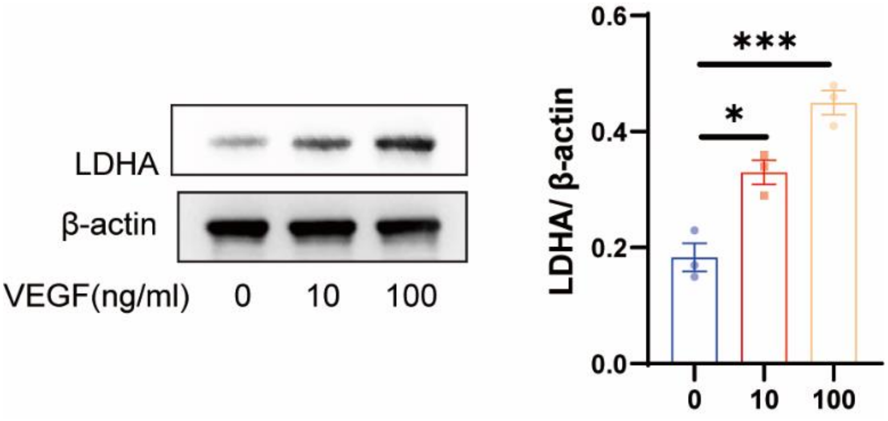

Fig S1. H3K9 lactylation is increased in endothelial cells in response to VEGF stimulation. Western blotting result of LDHA in HRMECs treated with different concentrations of VEGF (mean ± SEM; n=3 samples per group; *P < 0.05, ***P < 0.001, one-way ANOVA, Bonferroni post-hoc test)

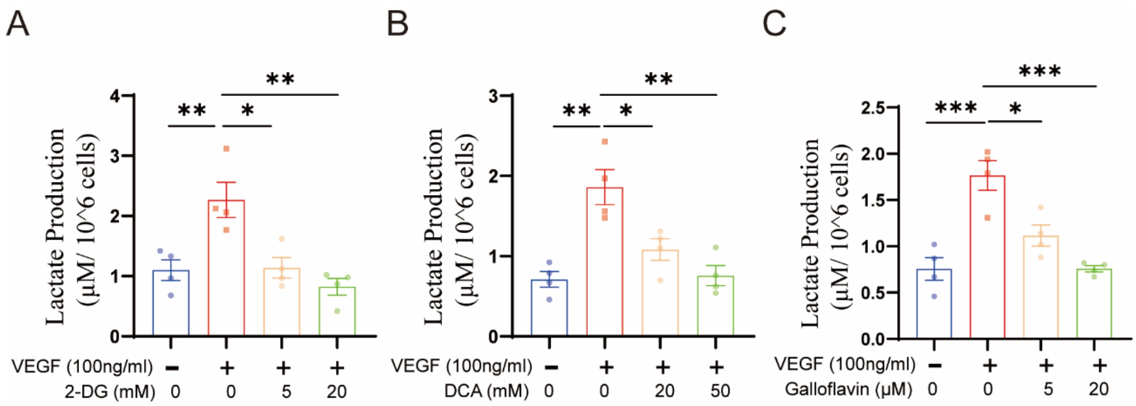

Fig S2. Inhibition of H3K9la inhibited angiogenesis in vivo and in vitro. A-C. The lactate content in HRMECs treated with glycolysis inhibitors as indicated mean ± SEM； n=4 samples per group; *P < 0.05, **P < 0.01, ***P < 0.001, one-way ANOVA, Bonferroni post-hoc test)

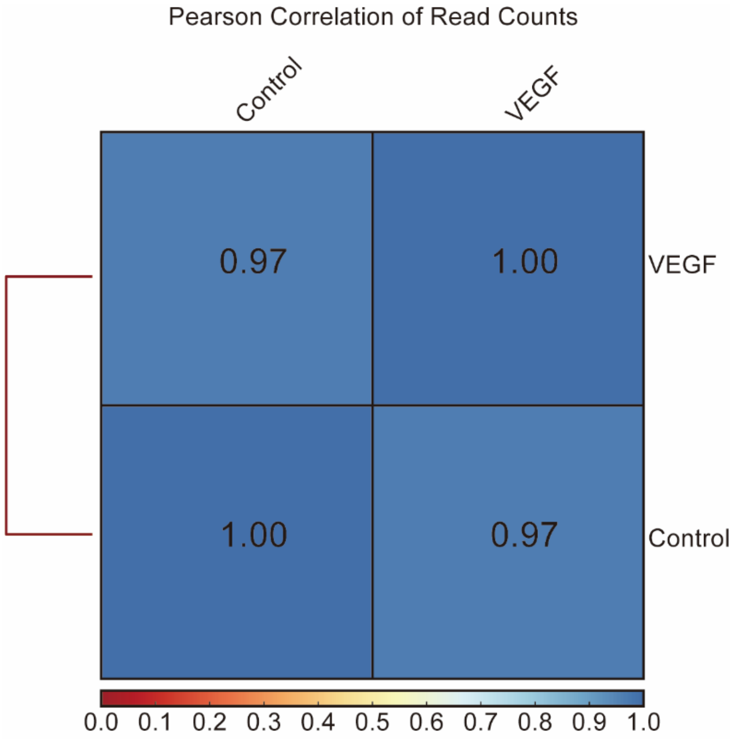

Fig S3. Pearson correlation of fold enrichment between two groups.

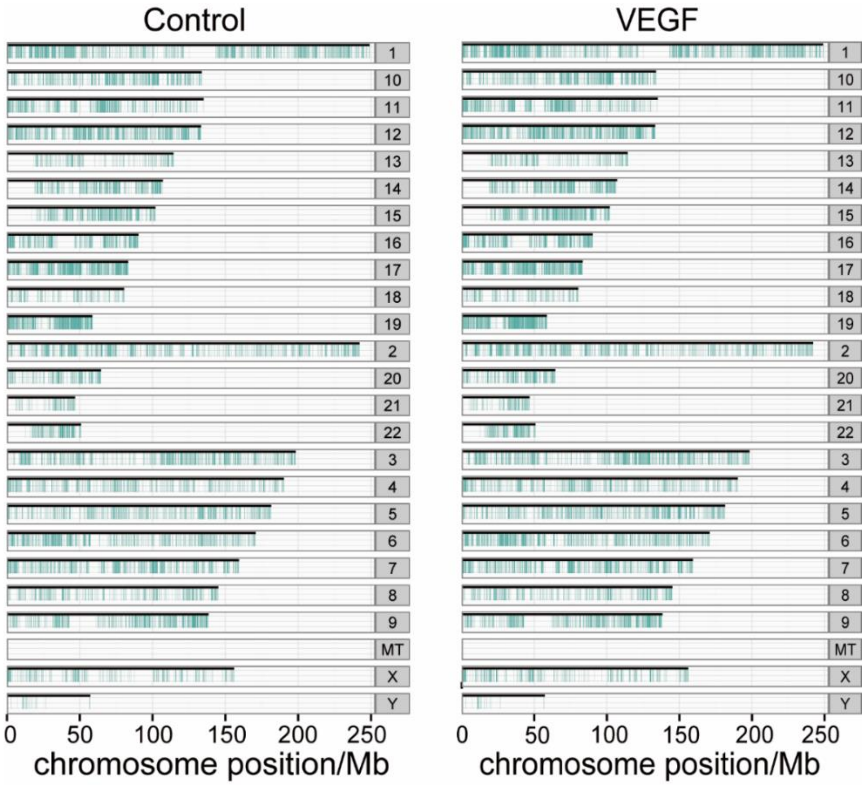

Fig S4. Location of identified peaks in chromosomes

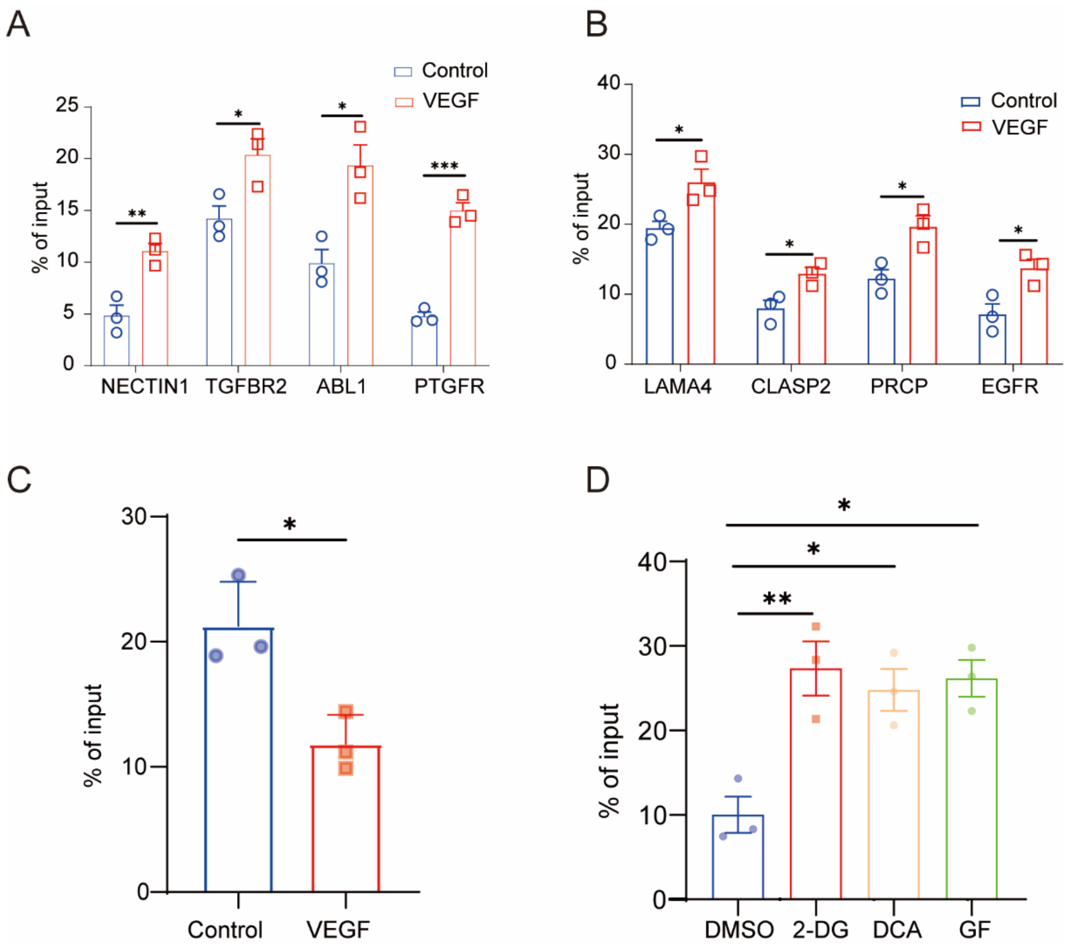

Fig S5. A feedback loop driven by H3K9 lactylation and HDAC2 promotes angiogenesis

A-B. CHIP qPCR analysis of the indicated promoters was conducted using anti-H3K9la antibodies in HRMECs under corresponding conditions (mean ± SEM; n=3 samples per group; *P < 0.05, **P<0.01, ***P < 0.001, unpaired Student's t-test)

C. CHIP qPCR revealed that hyperlactylation inhibited H3K9la's binding to the promoters of HDAC2 (mean ± SEM; n=3 samples per group; *P < 0.05, unpaired Student's t-test)

D. CHIP qPCR revealed that glycolysis inhibitors promoted H3K9la's binding to the promoters of HDAC2 (mean ± SEM; n=3 samples per group; *P < 0.05, **P<0.01, unpaired Student's t-test)
