# Supplementary material for: A feedback loop driven by H3K9 lactylation and HDAC2 in endothelial cells regulates VEGF-induced angiogenesis
Source: Genome Biol. 2024 Jun 25;25:165. doi: 10.1186/s13059-024-03308-5 (PMC11197246; doi:10.1186/s13059-024-03308-5)
Supplement: Supplementary file 2 — Additional file 2: Table S1 and Table S2. Primers used in this study. [file 13059_2024_3308_MOESM2_ESM.pdf]

Table S1. The primers for qRT-PCR assays

qRT-PCR Primers	
**Human**	
NECTIN1	5′-GCTACAGCAAGGCAGGCATCC-3′(forward)
	5′-TCCTCCTCCTCCTCCTCCTCATAG-3′(reverse)
TGFBR2	5′-TGCCAACAACATCAACCACAACAC-3′(forward)
	5′-TGCCACTGTCTCAAACTGCTCTG-3′(reverse)
ABL1	5′-AGCAACTACATCACGCCAGTCAAC-3′(forward)
	5′-CTCTCACTCTCACGCACCAAGAAG-3′(reverse)
PTGFR	5′-CAGCAGCACAGACAAGGCAGATC-3′(forward)
	5′-GGAGACACACATTATCGCCAGGAG-3′(reverse)
LAMA4	5′-AACCAGACTCAGTGATGCCGTTAAG-3′(forward)
	5′-CATCGTCGTCCTGTTGGCTTCC-3′(reverse)
CLASP2	5′-TGGCGATTCAATGTGTGGTGGTC-3′(forward)
	5′-GAGAGCGTGGAGAGGAGTGAGTAG-3′(reverse)
PRCP	5′-TGGTCAGGAGGTGGAGTAACTAAGG-3′(forward)
	5′-CTAACTTCCAAGGAGCGGGCTAAC-3′(reverse)
EGFR	5′- AGGCACGAGTAACAAGCTCAC-3′(forward)
	5′- ATGAGGACATAACCAGCCACC-3′(reverse)
HDAC2	5′-CGAGCATCAGACAAGCGGATAGC-3′(forward)
	5′-CGACCTCCTTCTCCTTCATCCTCAG-3′(reverse)

Table S2. The primers for CHIP-qPCR assays

CHI-qPCR Primers	
**Human**	
NECTIN1	5′-ATTCTCCATCCCGCCCTCTCC-3′(forward)
	5′-TGACAAAGGCAGGCAGTGACC-3′(reverse)
TGFBR2	5′-CCATCTGCAACCTCTGTCTCCTG-3′(forward)
	5′-TTGATGGTGCGTGCCTGTAGTC-3′(reverse)
ABL1	5′-TGCTTCCTTACCACTCACCTTGC-3′(forward)
	5′-GGTCCCACTGCCTGCGAAAG-3′(reverse)
PTGFR	5′-CCAATGCATCGGCTTCGCTTATC-3′(forward)
	5′-TCCAAACTCAGTGTCAGGCTCAG-3′(reverse)
LAMA4	5′-CCTGGAAGAGCACTACTGGATGTC-3′(forward)
	5′-GAGGAGCCACAGAGGCAGAAC-3′(reverse)
CLASP2	5′-AGGCAGCCAGTACAAGGAGAAAC-3′(forward)
	5′-AAAAGTCTGGGGTCTTGCTGAAAAC-3′(reverse)
PRCP	5′-TCCACTAGGCACTACTCTGACAGG-3′(forward)
	5′-CTTCCTTTCCACCACTTTCGAGAAC-3′(reverse)
EGFR	5′- GGGACCCTGGCACAGATTTGG-3′(forward)
	5′- TTAATTTCCGAGAGGGGCGTTCC-3′(reverse)
HDAC2	5′-AGCAGCAGCAGGAGGAGGAG-3′(forward)
	5′-GCCGCTCACCGTCGTAGTAG-3′(reverse)
