# Supplementary material for: A feedback loop driven by H3K9 lactylation and HDAC2 in endothelial cells regulates VEGF-induced angiogenesis
Source: Genome Biol. 2024 Jun 25;25:165. doi: 10.1186/s13059-024-03308-5 (PMC11197246; doi:10.1186/s13059-024-03308-5)

Fig. 1E

Pan-Kla

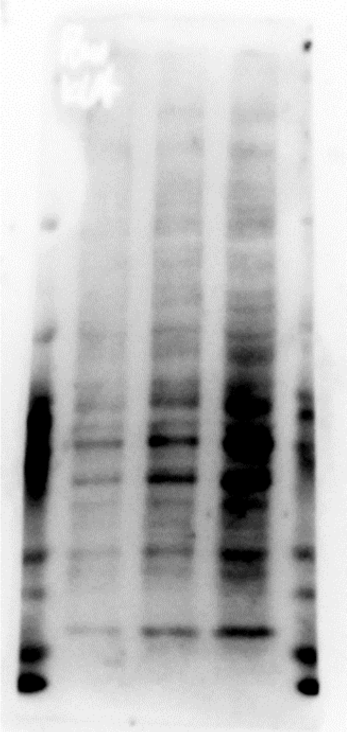

H4K5la

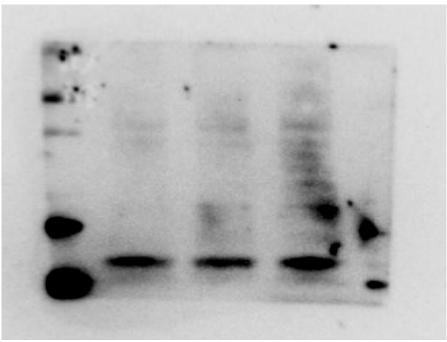

H4K12la

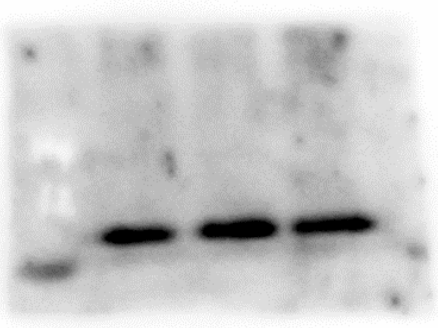

H4

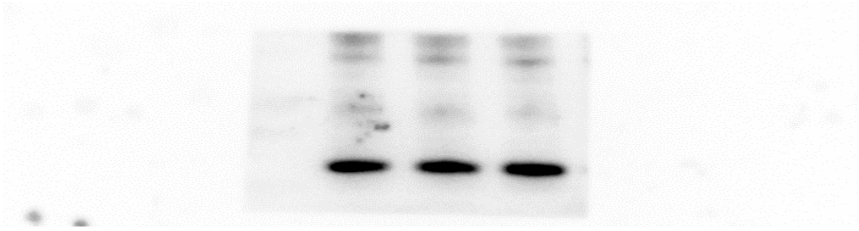

H3K9la

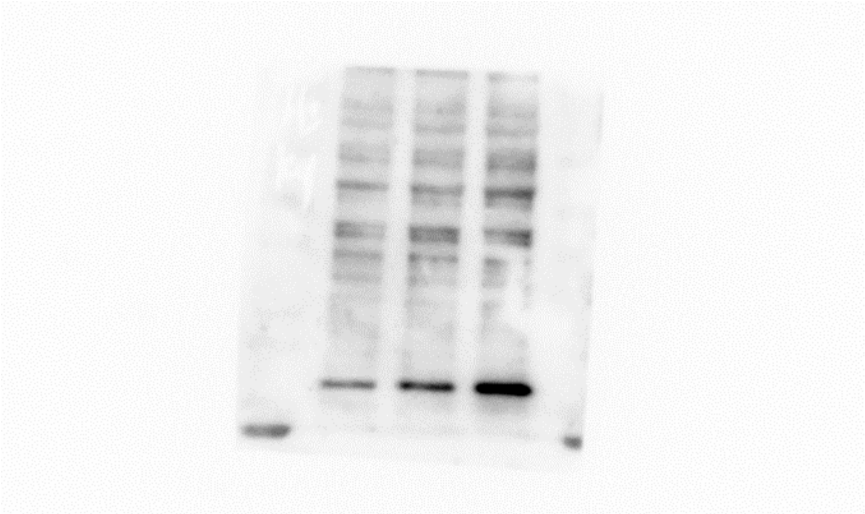

H3K14la

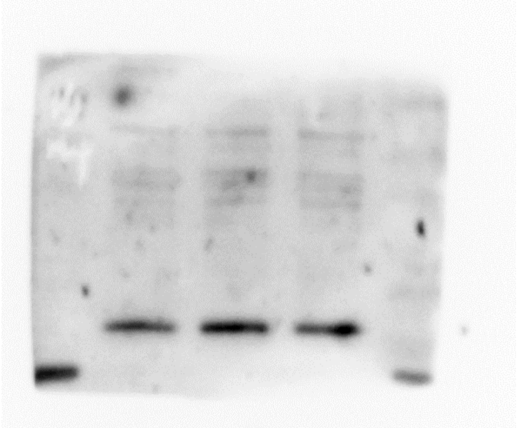

H3K18

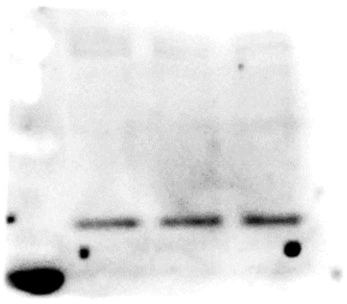

H3

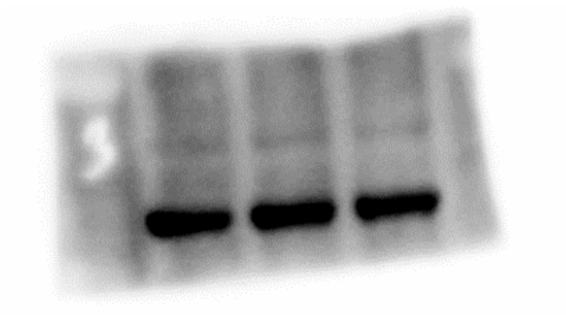

β-actin

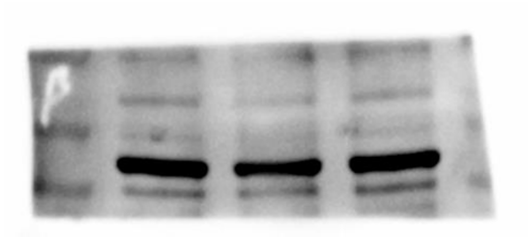

Fig 1F

H3K9la

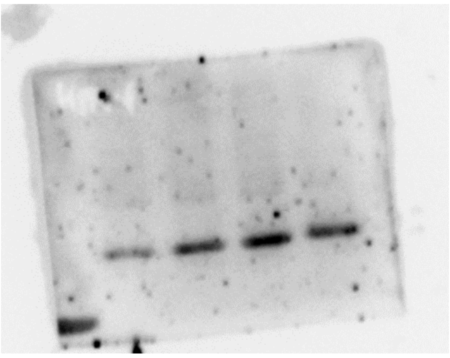

H3

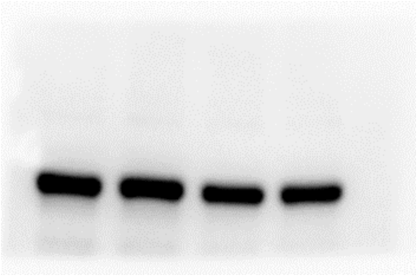

β-actin

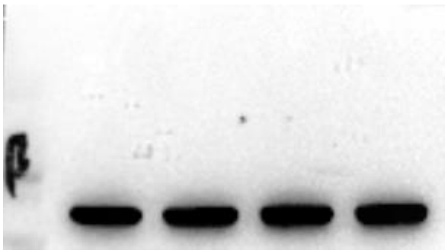

Fig 2B

H3K9la

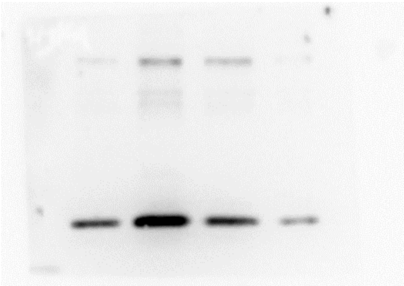

H3

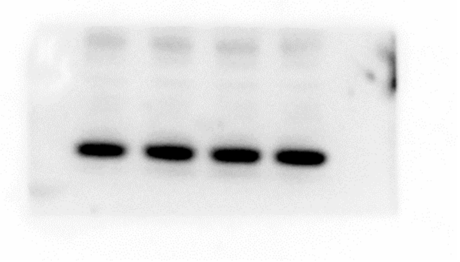

β -actin

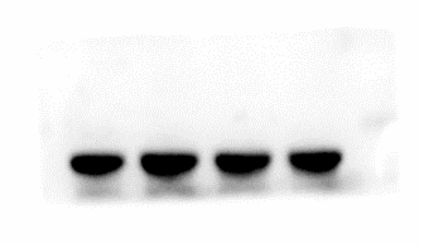

Fig. 2C

H3K9la

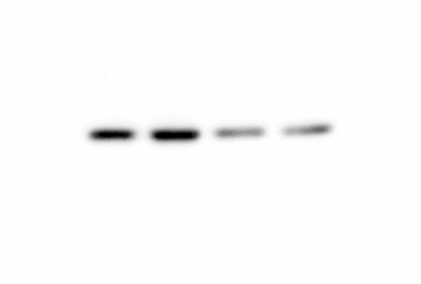

H3

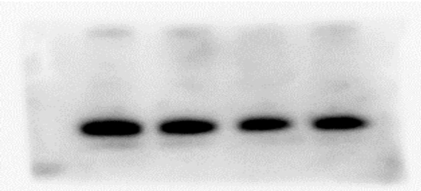

β-actin

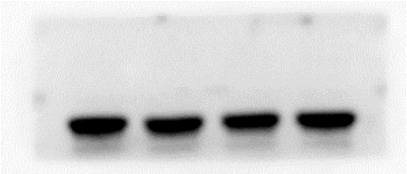

Fig. 2D

H3K9la

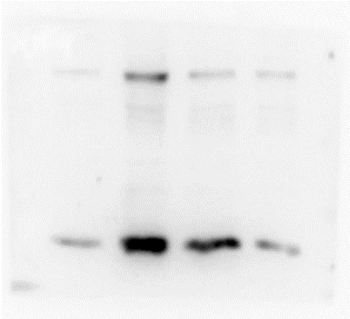

H3

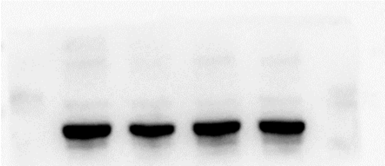

β-actin

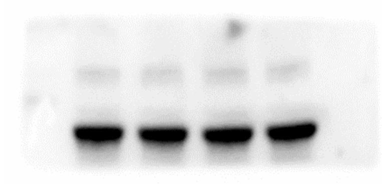

Fig. 2H

H3K9la

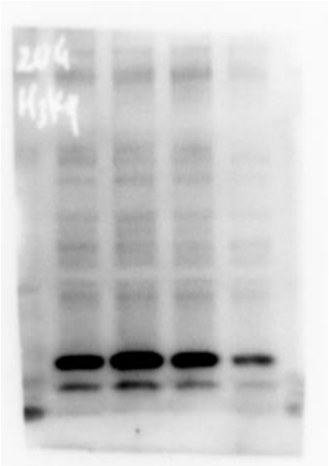

H3

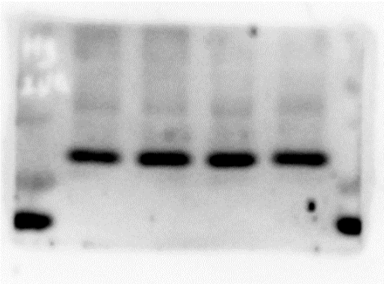

β-actin

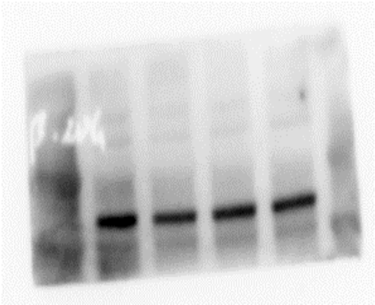

Fig. 2I

H3K9la

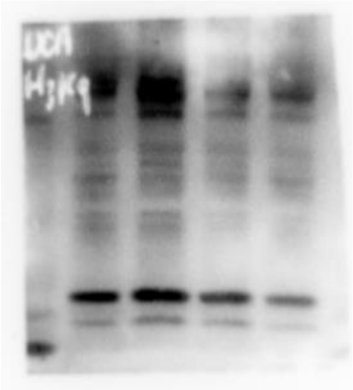

H3

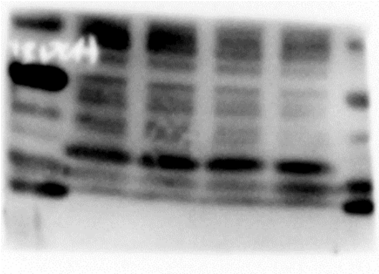

β-actin

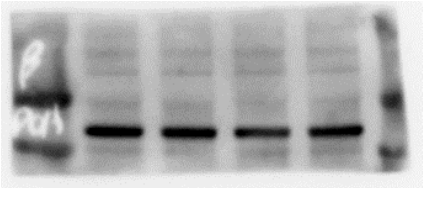

Fig. 2J

H3K9la

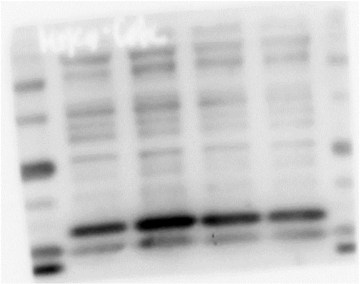

H3

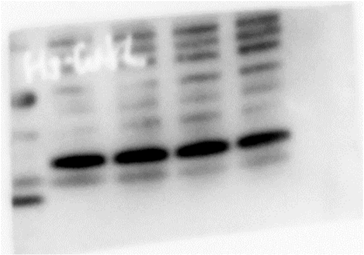

β-actin

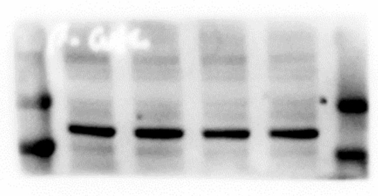

Fig. 5C

HDAC2

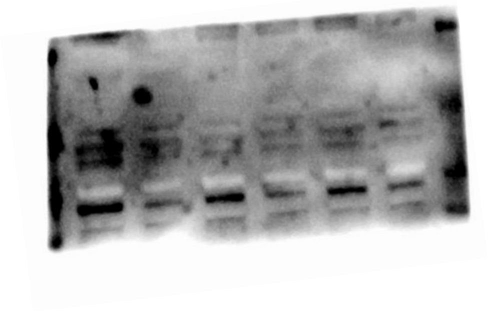

β-actin

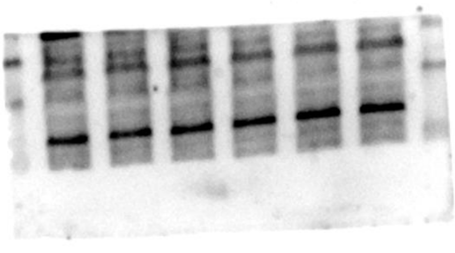

Fig. 5F

H3K9la

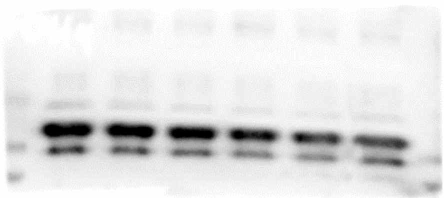

H3

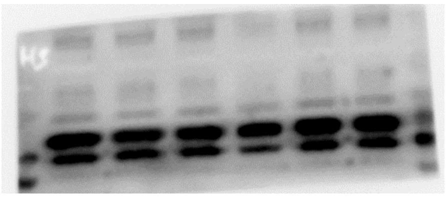

HDAC2

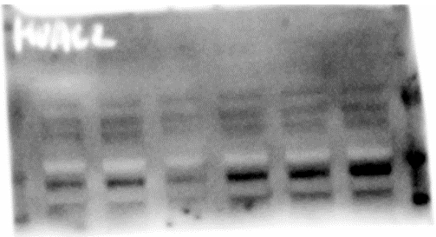

β-actin

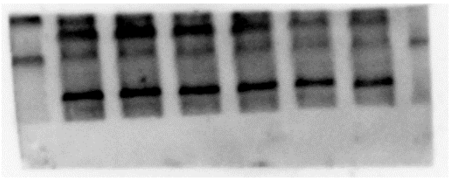

Fig. S1

LDHA

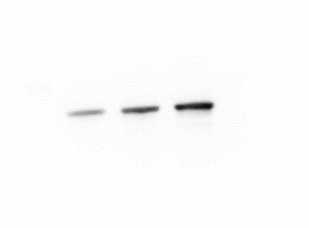

β-actin

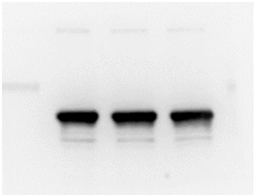

Supplement: Supplementary file 3 — Additional file 3. Uncropped blot images. [file 13059_2024_3308_MOESM3_ESM.pdf]
