# Supplementary material for: A feedback loop driven by H3K9 lactylation and HDAC2 in endothelial cells regulates VEGF-induced angiogenesis
Source: Genome Biol. 2024 Jun 25;25:165. doi: 10.1186/s13059-024-03308-5 (PMC11197246; doi:10.1186/s13059-024-03308-5)
Supplement: Supplementary file 4 — Additional file 4. Review history. [file 13059_2024_3308_MOESM4_ESM.docx]

Review History

**First round of review**

**Reviewer 1**

**Were you able to assess all statistics in the manuscript, including the appropriateness of statistical tests used?**

Yes, and I have assessed the statistics in my report.

**Comments to author:**

This work by Fan et al. examines the role of lactate-derived lactylation in VEGF-induced angiogenesis. The authors found that the lactylation levels of histone H3 at lysine 9 (H3K9) were significantly upregulated in VEGF-stimulated endothelial cells. Hyperlactylation promoted angiogenesis and its inhibition alleviated retinal neovascularization in an animal model of oxygen-induced retinopathy (OIR). The innovation of this study lies in exploring the role of H3K9 lactylation with CUT& Tag analysis. The authors demonstrate that H3K9 lactylation promotes the expression of a set of pro-angiogenic genes and there exists a feedback loop driven by H3K9 lactylation and HDAC2 that regulates angiogenesis. This study is important, highly novel, and of broad interest to genome science.

Below are some questions.
1. Introduction: This study focuses on the role of H3K9 lactylation in VEGF-induced angiogenesis. Histones (such as H3) do play important roles in multiple cellular processes (PMID：35338361; PMID：35794091), and it should be introduced and referenced in the Introduction section.
2. The authors declared that the production of lactate increased in ECs in response to VEGF stimulation (Figure 1D). Are there differences for lactate dehydrogenases (such as LDHA) in VEGF-treated ECs?
3. The authors showed that glycolysis inhibitors decreased the lactylation level in ECs (Figure 2 A-D). Does it correlate with a downregulation of lactate production?
4. Why does the lactylation level drop at day 25? Does it correlate with the severity of the OIR? This should be discussed.
5. The authors detected the effect of different concentrations of 2-DG, DCA and Galloflavin in Figure 2 B-D. What were the concentrations used in Figure2 E-G?
6. The fluorescence intensity of Dapi is too weak in the proliferation assays (Figure 1B; Figure 2F; Figure 5J). It's hard to count the total cell number. Please provide better images.
7. Using CUT& Tag analysis, the authors declare that the enrichment of H3K9la is different at the promoters of several angiogenic genes in VEGF-treated ECs. Please provide the quantitative values of enrichment scores in Control and VEGF groups in Figure 4A-D.
8. Line 124: 'Genome-wide CUT& Tag analysis is a newly described method for studying protein-DNA interactions. References are needed.
9. Methods: The item number of several reagents are missed (e.g. the PrimeScript qRT-PCR kit for RT-qPCR). Please check the whole manuscript and add them.
10. Glycolysis inhibitors are applied for in vivo experiments. But the detailed experimental methods are missed. Was it through intraperitoneal injection or gavage? What was the dosing frequency?
11. Line 213: "existsed" should be "existed'. Please check the spelling and grammar of the entire article.
12. The authors give the "n" in Figure legends but don't explain what it means. Is it number of samples or number of technical replicates?
13. Limitations of this study should be discussed in the Discussion section.

**Reviewer 2**

**Were you able to assess all statistics in the manuscript, including the appropriateness of statistical tests used?**

Yes, and I have assessed the statistics in my report.

**Comments to author:**

In this manuscript, authors identified the H3K9 lactylation in endothelial cells as an important biological event for pathological angiogenesis. Mechanically, they claimed an VEGF/H3K9la/HDAC2 feedback loop on endothelial cells' angiogenic activity. Overall, this is an interesting work for illustrating the functioning of VEGF-mediated glycolysis in the regulation of angiogenesis. However, I have following concerns.
1. H3K9la in control group in Figure 1G is not consistent with immunoblot results. Does H3K9la only appear upon pathological stimuli or also exist during regular angiogenesis?
2. The quality of retina images needs to be improved. High-power of representative areas helps demonstrate the details of angiogenesis. It would be better to include the quantification of avascular areas.
3. An important missing piece is the molecular mechanism to illustrate how H3K9 lactylation regulates the level of HDAC2.
4. It is kind of weird that the major targets of H3K9lac only include a few angiogenesis related genes but affect the pathological angiogenesis significantly. How about the effect of H3K9lac on the key angiogenic genes such as VEGFR2, FGFR1 and other correlated RTKs?
5. How were representative images/figures chosen? Please note the approach used to select representative images in the main text or figure legends.
6. Some tests (eg t-tests, ANOVA) used assume normality, however it is not clear how normality was established. Please note that common tests of normality are not powered to detect departures from normality when n is small (eg n<6) and in these cases normality should be support by external information or non-parametric tests should be used. Also, when repeated comparisons against a control group were performed, multiple test corrections should be applied and indicated in figure legends.

**Authors Response**

**Point-by-point responses to the reviewers’ comments:**

**Reviewer #1**:

This work by Fan et al. examines the role of lactate-derived lactylation in VEGF-induced angiogenesis. The authors found that the lactylation levels of histone H3 at lysine 9 (H3K9) were significantly upregulated in VEGF-stimulated endothelial cells. Hyperlactylation promoted angiogenesis and its inhibition alleviated retinal neovascularization in an animal model of oxygen-induced retinopathy (OIR). The innovation of this study lies in exploring the role of H3K9 lactylation with CUT& Tag analysis. The authors demonstrate that H3K9 lactylation promotes the expression of a set of pro-angiogenic genes and there exists a feedback loop driven by H3K9 lactylation and HDAC2 that regulates angiogenesis. This study is important, highly novel, and of broad interest to genome science.

Below are some questions.

1. Introduction: This study focuses on the role of H3K9 lactylation in VEGF-induced angiogenesis. Histones (such as H3) do play important roles in multiple cellular processes (PMID：35338361; PMID：35794091), and it should be introduced and referenced in the Introduction section.

***Response: We appreciate the reviewer’s suggestions. We have added information about histones in the Introduction section (Please refer to page 3 lines 67-78，references 14-23).***

2. The authors declared that the production of lactate increased in ECs in response to VEGF stimulation (Figure 1D). Are there differences for lactate dehydrogenases (such as LDHA) in VEGF-treated ECs?

***Response: We appreciate the reviewer’s comments. We detected the expression of LDHA in VEGF-treated ECs and found that it’s upregulated in ECs in response to VEGF stimulation. These results further indicated that VEGF treatment promoted glycolysis in endothelial cells (Please refer to page 4 lines 98-100, Fig. S1).***

3. The authors showed that glycolysis inhibitors decreased the lactylation level in ECs (Figure 2 A-D). Does it correlate with a downregulation of lactate production?

***Response: We appreciate the reviewer’s comments. We detected the lactate content in glycolysis inhibitors-treated endothelial cells and observed that the lactate content was decreased in response to treatment. Therefore, the decreased lactylation levels in ECs* *correlate with downregulation of lactate production (Please refer to page 5 lines 119-121, Fig. S2A-C).***

4. Why does the lactylation level drop at day 25? Does it correlate with the severity of the OIR? This should be discussed.

***Response: We appreciate the reviewer’s comments. Yes, the lactylation level correlates the severity of OIR. As previous studies from our lab and others reported (PMID: 33586674; PMID: 35895036; PMID: 37085894), the neovascularization of OIR reaches its peak around P17 and alleviated at day 25. Therefore, the lactylation level was associated with the severity of the OIR. We have added information to discuss this result (Please refer to page 4 lines 106-108).***

5. The authors detected the effect of different concentrations of 2-DG, DCA and Galloflavin in Figure 2 B-D. What were the concentrations used in Figure2 E-G?

***Response: We appreciate the reviewer’s comments. We have added the concentrations of glycolysis inhibitors in Figure2 E-G.***

6. The fluorescence intensity of Dapi is too weak in the proliferation assays (Figure 1B; Figure 2F; Figure 5J). It's hard to count the total cell number. Please provide better images.

***Response: We appreciate the reviewer’s comments. We have provided better images (Please refer to Figure 1B; Figure 2F; Figure 5J).***

7. Using CUT& Tag analysis, the authors declare that the enrichment of H3K9la is different at the promoters of several angiogenic genes in VEGF-treated ECs. Please provide the quantitative values of enrichment scores in Control and VEGF groups in Figure 4A-D.

***Response: We appreciate the reviewer’s comments. We have provided the quantitative values of enrichment scores in Control and VEGF groups in Figure 4A-D.***

8. Line 124: 'Genome-wide CUT& Tag analysis is a newly described method for studying protein-DNA interactions. References are needed.

***Response: We appreciate the reviewer’s comments. We have added the reference (Please refer to page 5 line 133, reference 28).***

9. Methods: The item number of several reagents are missed (e.g. the PrimeScript qRT-PCR kit for RT-qPCR). Please check the whole manuscript and add them.

***Response: We appreciate the reviewer’s comments. We have checked the whole manuscript and added item numbers (Please refer to page 7 line 270; page 7 line 285).***

10. Glycolysis inhibitors are applied for in vivo experiments. But the detailed experimental methods are missed. Was it through intraperitoneal injection or gavage? What was the dosing frequency?

***Response: We appreciate the reviewer’s comments. The glycolysis inhibitors are applied by intravitreal injection. We have added the detailed protocols in Methods section (Please refer to page 7 lines 255-256).***

11. Line 213: "existsed" should be "existed'. Please check the spelling and grammar of the entire article.

***Response: We appreciate the reviewer’s comments. We have checked the spelling and grammar of the entire article.***

***“was” to “is” (Please see page 2, line 49)***

***“behavior” to “behaviors” (Please see page 3, line 57)***

***“was” to “has been” (Please see page 3, line 62)***

***“existsted” to “existed” (Please see page 8, line 225)***

***“investigated” to “investigate” (Please see page 8, line 231)***

12. The authors give the "n" in Figure legends but don't explain what it means. Is it number of samples or number of technical replicates?

***Response: We appreciate the reviewer’s comments. We have added information to state each “n” stands in figure legends.***

13. Limitations of this study should be discussed in the Discussion section.

***Response: We appreciate the reviewer’s comments. We have added information about the limitations of this study (Please refer to page 8 lines 233-240).***

**Reviewer #2**:

In this manuscript, authors identified the H3K9 lactylation in endothelial cells as an important biological event for pathological angiogenesis. Mechanically, they claimed an VEGF/H3K9la/HDAC2 feedback loop on endothelial cells' angiogenic activity. Overall, this is an interesting work for illustrating the functioning of VEGF-mediated glycolysis in the regulation of angiogenesis. However, I have following concerns.

1. H3K9la in control group in Figure 1G is not consistent with immunoblot results. Does H3K9la only appear upon pathological stimuli or also exist during regular angiogenesis?

***Response: We appreciate your comments. According to our results, we observed that H3K9la also appear in regular angiogenesis. As for why H3K9la in control group in Figure 1G seems not consistent with immunoblot results, we think it’s partly due to the limitations of immunofluorescence assays. The lactylation level in control group was largely lower than OIR group. Images from different groups were captured under same parameters. Due to the high fluorescence intensity of the OIR group, we set the exposure time at a reasonable value to avoid overexposure, which resulted in the control group appearing to have a very low fluorescence intensity. In addition, during our first submission, all figures were embedded in one single PDF file and then submitted, which leads to poor clarity of these images. To avoid this problem, we have resubmitted separate figures. And it can be seen that H3K9la also appear in regular angiogenesis (Please refer to Fig. 1G).***

2. The quality of retina images needs to be improved. High-power of representative areas helps demonstrate the details of angiogenesis. It would be better to include the quantification of avascular areas.

***Response: We appreciate your comments. We have provided images with better quality and added high-power images of representative areas. We quantified the avascular area and found that there no significant differences between groups. During the induction of OIR, the mice were exposure to hyperoxia (75%O_2_) between P7-P12, which leads to vascular blockage and loss, and then returned to normal air environment. The inhibitors were applied at P13 at neovascularization stage. Therefore, we think that inhibiting histone lactylation only influenced retinal neovascularization, but not vascular loss (Please refer to Fig. 2K, page 4 lines 124-126).***

3. An important missing piece is the molecular mechanism to illustrate how H3K9 lactylation regulates the level of HDAC2.

***Response: We appreciate your comments. As previous studies reported, histones play important roles in modulating chromatin structure and function, including* *transcriptional regulation (PMID: 35338361; PMID: 16261189). Post-translational modifications could influence histone’s binding to the promoters of specific genes and then regulates gene transcription (PMID: 33726814; PMID: 35303422). In our study, CUT & Tag analysis showed that H3K9la directly binds to the promoter of HDAC2. We added experiments and further found that hyperlactylation of H3K9 inhibited its interaction with HDAC2 by CHIP-qPCR assays, and this process is reversed in response to glycolysis inhibitors treatment. Therefore, we think that lactylation regulates H3K9’s binding to the promoter of HDAC2 and modulates its expression (Please refer to page 6 lines 168-170, Fig. S5 C-D).***

4. It is kind of weird that the major targets of H3K9lac only include a few angiogenesis related genes but affect the pathological angiogenesis significantly. How about the effect of H3K9lac on the key angiogenic genes such as VEGFR2, FGFR1 and other correlated RTKs?

***Response: We appreciate your comments. In the current study, we found that hyperlactylation of H3K9 influenced the expression of a set of angiogenic genes including TGFBR2, PRCP, EGFR etc. Thanks for your kind reminding, we further explored the effect of H3K9la on the classical angiogenic genes including VEGFR1, VEGFR2, VEGFR3, FGFR1, FGFR2, PDGFRA and PDGFRB. However, we found that there are no significant differences of H3K9la enrichment in these genes (R-Fig.1). The regulation of gene expression is a sophisticated process. We supposed that not H3K9la but other transcription factors or histone modifications may play a function in modulating these gene’s expression. Further investigations are warranted to elucidate these mechanisms and this is discussed in the limitations of our study (Please refer to page 6 lines 233-240).***

**R-Fig. 1 Genome browser tracks of CUT& Tag signal at the indicated genes**

5. How were representative images/figures chosen? Please note the approach used to select representative images in the main text or figure legends.

***Response: We appreciate your comments. For tube formation, transwell and EdU assays, we captured*** ***four random images for each sample and the average value is used for statistical analysis. We added contents to explain this issue in the Methods section (Please refer to page 9 lines 261-282).***

6. Some tests (eg t-tests, ANOVA) used assume normality, however it is not clear how normality was established. Please note that common tests of normality are not powered to detect departures from normality when n is small (eg n<6) and in these cases normality should be support by external information or non-parametric tests should be used. Also, when repeated comparisons against a control group were performed, multiple test corrections should be applied and indicated in figure legends.

***Response: We appreciate your comments. We used*** ***Shapiro-Wilk test to test the normality of data. Comparisons between two groups were performed using an unpaired Student’s t-test or Mann-Whitney U test according to its normality. One-way ANOVA,*** ***Bonferroni post-hoc test was applied to multiple groups. The detailed information of statistical analysis was indicated in the figure legends.***

**Second round of review**

**Reviewer 1**

The authors have addressed all my concerns and I have no further questions. I recommend the publication of this important paper.

**Reviewer 2**

The authors have addressed most of the concerns properly. It is recommended to provide further discussions about the molecular mechanism to illustrate how H3K9 lactylation regulates the level of HDAC2 in the "Discussion" part, according to your summary in the #3 response of Reviewer 2.

**Authors Response**

**Point-by-point responses to the reviewers’ comments:**

**Reviewer 2**

The authors have addressed most of the concerns properly. It is recommended to provide further discussions about the molecular mechanism to illustrate how H3K9 lactylation regulates the level of HDAC2 in the "Discussion" part, according to your summary in the #3 response of Reviewer 2.

*Response: We have added further discussions about the molecular mechanism to demonstrate how H3K9 lactylation regulates HDAC2 level in the "Discussion" section.*
